# Comparison of home-based palliative care delivered by community health workers versus usual care: research protocol for a pilot randomized controlled trial

**DOI:** 10.1186/s12904-023-01235-z

**Published:** 2023-09-02

**Authors:** Suparna Qanungo, Kathleen B. Cartmell, Martina Mueller, Melissa Butcher, Saswati Sarkar, Tyler-Gail Carlson, Mohan Madisetti, Gaurav Kumar

**Affiliations:** 1 College of Nursing, Medical University of South Carolina, 99 Jonathan Lucas Street., MSC 160, Charleston, SC 29425-1600 USA; 2 Department of Public Health Sciences, Clemson University, Clemson, SC USA; 3 TATA Medical Center, Kolkata, West Bengal India; 4 TATA Medical Center, Rajarhat, Kolkata India

**Keywords:** Palliative care, Cancer, Community health workers, Home-based care, randomized controlled trial

## Abstract

**Background:**

Research studies demonstrate that palliative care can improve patient outcomes such as quality of life, symptom burden and patient satisfaction with care (Gomes B, et al. Effectiveness and cost-effectiveness of home palliative care services for adults with advanced illness and their caregivers. Cochrane Database Syst Rev. 2013(6):CD00776) (World Health Organization. Palliative Care. Published 2020.). While 76% of patients who need palliative care live in limited-resource countries, access to high quality palliative services in these countries is minimal (Worldwide Hospice and Palliative Care Association and World Health Organization. Global Atlas of Palliative Care (2nd ed). 2020.). In 2014 the Worldwide Hospice Palliative Care Alliance, with strong endorsement by the WHO, released the Palliative Care Toolkit to provide a training and implementation toolkit for empowering community members to deliver palliative care in resource poor settings (Worldwide Hospice and Palliative Care Association and World Health Organization. Global Atlas of Palliative Care at the End of Life. Geneva, Switzerland 2014.). They encouraged researchers and public health practitioners to conduct rigorous evaluation of the toolkit in diverse settings and contexts. To address this need, we will conduct a pilot randomized controlled trial (RCT) to examine implementation and explore potential effect of an intervention based upon the Palliative Care Toolkit, as adapted and used by community health workers (CHWs) working with a cancer center in Kolkata, India to deliver home-based palliative care for rural patients.

**Methods:**

Utilizing a randomized controlled trial design, intervention patients (n = 45) receive home-based palliative services (Pal-Care) delivered by community health workers (CHWs), with comparison against a control group of patients (n = 45) who receive usual cancer-center-based palliative services. Primary outcome measures include evaluation of CHW training outcomes, roles and responsibilities of the CHWS and how they assist patients, trial recruitment, stakeholder perceptions of the intervention, and fidelity to study protocol. Secondary outcomes measure patient self-report of health-related quality of life, symptom burden, palliative needs and patient care experience, outcomes The RE-AIM framework guides our evaluation plan to measure the reach, effectiveness, adoption, implementation and maintenance of the Pal-Care intervention (Gaglio B, et al. The RE-AIM framework: a systematic review of use over time. Am J Public Health. 2013;103(6):e38?46.). Data will be analyzed in SAS. All measures will be evaluated overall and by patient age, gender and cancer type and by CHW caseload.

**Discussion:**

Pal-Care is a RCT funded by the NCI to explore utilization of CHWs to deliver a home-based palliative care intervention built upon the WHO Palliative Care toolkit (PCT), as compared to a usual care control group. The long-term goal of this research is to develop an effective and sustainable model for delivering home-based palliative care for cancer patients in underserved areas.

**Trial registration (TRN):**

ClinicalTrials.gov ID# NCT04972630.

## Introduction

### Background and rationale

Approximately 80% of patients who need palliative care to control pain and suffering at the end of life live in low to middle income countries (LMICs), but only about 14% of these patients receive services [1]. There is also a lack of rigorous research to inform best practices in palliative care in LMICs, with most palliative care practices based upon research conducted in high income countries such as England and the United States [2]. Palliative care can reduce pain and symptom burden [3, 4], increase QOL [4, 5], satisfaction with care [6, 7], and the likelihood of dying at home [3, 6]. At a health-system level, palliative care can reduce hospitalizations [6, 8], ED visits [6], and healthcare costs [6]. Despite strong evidence for the benefits of palliative care, a myriad of contextual factors make its delivery challenging in low and middle income countries (LMIC) such as India. Palliative care use in low resource settings is extremely limited, while its demand is increasing and exacerbates suffering in patient with life-limiting illnesses such as cancer [2, 9]. This is particularly problematic for those living in rural areas of the world where culture and cost restrict use of palliative care strategies. Barriers for cancer patients in India include: limited oncologists for a vast rural population, travel distance, treatment is costly and only about 20% of India has health insurance, laws regulate the distribution of morphine making it difficult for patients to obtain, and cultural factors such as the belief that cancer is contagious and the desire to not burden one’s family discourage patients from seeking palliative care [10, 11]. Due to these many factors, most patients end up dying at home without access to care or basic pain management.

In 2014 the WHO and Worldwide Hospice Palliative Care Alliance released the Palliative Care Toolkit to provide a training and implementation toolkit for empowering community members to deliver palliative care in resource poor settings [12]. The premise of the toolkit is that basic, effective palliative care can be delivered within existing community and health infrastructure by people without specialized training. The toolkit provides educational materials and data collection tools for providing palliative care, which mirror the WHO definition of key palliative care components [12]. Toolkit materials include a symptom control guide and protocols for using palliative medications, forms for patient records, teaching aids and advocacy materials. In 2015, an evaluation of the toolkit was published, which reports on its utilization in 43 countries (46% in Africa, 29% in South Asia, 16% in South America), reflecting its global diffusion. Over 90% of respondents felt the toolkit improved their understanding of palliative care, enhanced their knowledge of implementing palliative care, and improved their confidence in case management. While the toolkit has not undergone extensive impact evaluation, it is founded on evidence-based palliative care principles [12]. In the Atlas of Palliative Care report, the WHO recommended research is urgently needed to evaluate use of the toolkit in diverse settings [12]. The synergy between the WHO global palliative care priorities and availability of the toolkit creates an optimal milieu to implement and evaluate a palliative care model for use in resource poor settings.

In our formative study, we identified CHWs as a robust workforce that can help to expand the reach of palliative services to rural patients. CHWs are embedded in communities throughout India and are often the only health providers in rural areas [13]. This workforce is often underutilized as rural healthcare partners by the medical community due to lack of formal medical training [6]. Additional research is needed to implement novel models to provide palliative care particularly in LMICs.

As recommended in the Palliative Care Toolkit, palliative care in low resource countries must be integrated within existing healthcare systems, but also utilize community members to expand the reach of scarce healthcare providers [12]. Thus, we propose to conduct a pragmatic clinical trial, with patient level randomization, to examine implementation and potential effect of the Palliative Care Toolkit, as adapted and used by CHWs working with cancer center to deliver home-based palliative care for rural patients. This study builds upon the Toolkit to include: use of community health workers to deliver the intervention, use of simple tele-health tools to overcome geographic barriers, incorporation of culturally-tailored education, and adaptation of toolkit materials to fit existing structures in a cancer center setting that serves poor, rural patients. Specifically, we will implement a novel method of utilizing community members trained as CHWs to deliver and monitor palliative care services to patients and their families and compare it to usual cancer-center based care. The long-term goal of this research is to.

develop a feasible, effective and sustainable model for delivering home-based palliative care for cancer patients in underserved areas of rural India. This model is likely to be adaptable for use with other life-threatening illnesses beyond cancer and in resource limited settings in other countries. This research may also provide clues into how community-based resources could be better leveraged to meet the needs of medically underserved rural patients in the developed countries.

### Objectives

This study will evaluate (1) the implementation of the home-based palliative care intervention (Pal-Care) in terms of standard implementation measures specified by the RE-AIM Framework. These include evaluation of intervention Reach, Effectiveness, Adoption, Implementation and Maintenance, and (2) the outcomes of the Pal-Care intervention to determine its relative effects compared to a standard usual care control group on diverse patient endpoints, including health-related quality of life, symptom burden, palliative care needs and experience with care.

### Trial design

**Fig. 1:**
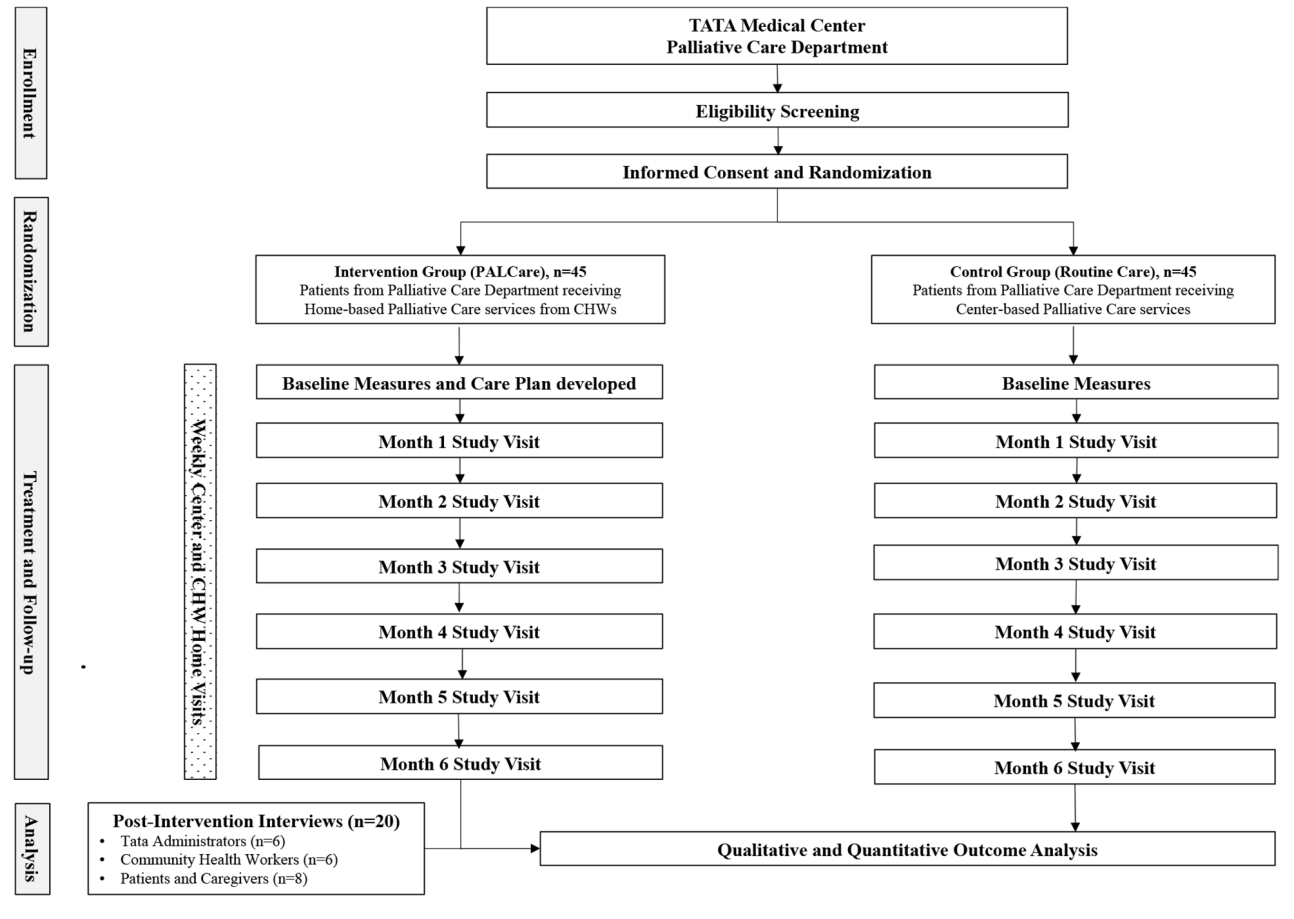
SPIRIT Diagram

This is a multi-site, randomized controlled trial. Patient’s from TATA Medical Center’s (TMC)  Palliative Care Unit will be screened for eligibility and consented to participate in the study. Patient participants will be randomly allocated to the intervention or control group via simple randomization. The intervention group will receive home-based palliative care delivered by CHWs and the control group will receive usual palliative care from TMC over a 6-month period. The trial protocol is elaborated in the SPIRIT diagram (Fig. 1).

## Methods

### Study setting

This study is a collaboration between the Medical University of South Carolina (MUSC), Clemson University and TATA Medical Center (TMC) in Kolkata, India. The study will be conducted among cancer patients in TMC’s Palliative Care Department (inpatient and outpatient), including those who receive palliative care in TMC’s “Premashraya” inpatient service that serves poor, uninsured patients.

### Eligibility criteria

#### Community health workers eligibility criteria

CHWs will be selected through an interview process if they met the eligibility criteria: (a) residing in the 24 Parganas Region where the Pal-Care intervention will be implemented, (b) 6–24 months of training from the Indian government or private institutions in allied medical field (e.g. diploma in health related field, ANM-auxiliary nursing midwifery diploma, associate degree/ diploma in social service).

#### Study participant eligibility criteria

Patients receiving care through TMCs Palliative Care Unit will be screened to determine study eligibility. Ninety terminally ill cancer patients from TMC’s Palliative Care Department who reside in the 24 Parganas region will be enrolled and randomized to the intervention or control group via simple randomization. Patient participant eligibility criteria includes: (a) age ≥ 18, any cancer type, (b) late-stage cancer, (c) residence in 24 Parganas, (d) documentation from medical doctor that the patient is to receive palliative care, (e) ECOG status of 0–3 and (f) patient willingness to participate in data collection. These broad eligibility criteria will be used, as the study will evaluate the Pal-Care intervention under real world conditions.

### Intervention

#### Community health worker training to deliver the intervention

The CHW team received didactic and experiential training prior to implementation of the intervention. The training was informed by our prior training of CHWs [10, 11, 14] and understanding the the complexity of skills required to deliver palliative care. First, CHWs participated in a 40-hour didactic/experiential training that covered the WHO Palliative Care Toolkit content: palliative care principles, intervention protocol, assessment and management of pain and other symptoms, care for caregivers, communication skills, patient support resources, strategies for team care, and cultural and spiritual considerations in end of life care. To reinforce didactic content, case studies from the toolkit were provided to CHWs to practice their new palliative skills. Case studies focused on essential palliative care scenarios related to physical, psychological, social and spiritual aspects. Second, CHWs shadowed clinical team members (palliative oncologists, nurses, social worker, psychologist) on patient encounters for 80 h. A pre-post training survey and skills checklist was administered to assess attainment of palliative care knowledge domains and assess palliative skills mastery.

#### Pal-Care intervention

As shown in Table 1, Intervention group participants will receive home based Pal-Care. Pal-Care begins with an appointment for the patient and caregiver to meet with their assigned CHW and the clinical team, which includes a Palliative oncologist, research coordinator, social worker, and nurse. At this appointment, patient participant’s baseline health and needs are assessed, and an individualized care plan is created for each participant. The CHW will make home visits to participants 1 + times weekly, depending on need. The CHW will use resources from the WHO Palliative Care Toolkit to: (1) monitor the patient’s health condition, (2) provide basic palliative care (i.e., medication administration, wound care, catheter care), (3) teach caregivers to deliver care and address patients’ concerns, (4) monitor pain and symptom control, and (5) assist patients to contact their oncologist or other resources when needed. CHWs will maintain care logs from the Palliative Care Toolkit including a caseload registry, a log of patient needs and services provided, longitudinal assessment of patient symptom scores, administration of medication, referrals to the cancer center and community resources, and monthly service reports. CHWs will travel to TMC on the participant’s behalf to obtain morphine to address the transportation barrier impacting the majority of participants. They will also educate patients and family members to dispel common myths about cancer and its treatment. The CHW and cancer center team will debrief weekly on patients’ status and plan of care for patients. CHWs will use tablets/android phones with a free platform, along with WhatsApp for CHWs to communicate with the Tata-based care team, as previous studies have identified mobile devices as effective ways to improve CHW performance in LMICs [15].

### Control

As shown in Table 1, the control group will receive usual care services in which the patient or caregiver must visit the TMC cancer center for care. TMC services include consultation with a multi-disciplinary team (Palliative oncologist, nurse, social worker and a psychologist), a 21-day morphine supply, basic training on medication usage, catheter, and wound care, other topics as relevant, and psychological counseling. Patients must return to the cancer center as needed for follow-up care and are provided with a 24/7 hotline to call in case of emergency. Intervention patients will receive TMC based consultation from a multi-disciplinary team similar to the control patients. Additionally, at the time of the initial consult they will be connected to a CHW who will provide home-based palliative care services as listed in Table 1.

**Table 1:** Control group versus Intervention group Care Processes

Care Component	Control Group	Intervention Pal-Care Group
Advanced care planning/ goal setting	Patients receive an initial consult with an oncologist, social worker, nurse, and psychologist.	Initial appointment is arranged for the patient and their caregiver to meet with their CHW, the oncologist, social worker, nurse, and psychologist to create an individualized care plan.
Emergency services	Patients are provided a 24/7 hotline to reach TMC.	Patients receive after hours emergency contact numbers.
Pain and symptom management	Received during visits to TMC	“Patient Pain Assessment Tool” is utilized weekly by CHW. Medication and non-pharmacological interventions are discussed with caregiver as needed. CHWs obtain morphine from TMC and bring the medication to patients’ homes.
Psychosocial and spiritual support	Basic psychological support provided at TMC if patients express feelings of psychological distress	Provided by CHW weekly, or as needed (if the CHW identifies or the patient express feelings of psychological distress), during each home visit
Caregiver support	Received at TMC visit.	Education is provided, and caregiver wellbeing is assessed and reinforced by CHW at each weekly home visit

### Recruitment

#### Community health workers recruitment

The TMC team will recruit and train 6 CHWs from the 24 Parganas Region where the Pal-Care will be implemented. The CHWs will be embedded in outlying areas around the cancer center, often represent the only providers in rural communities, and tend to be trusted in their communities. The CHWs will all be recruited through an interview process where their potential to care for basic medical problems will be evaluated.

#### Study participant recruitment

All study recruitment and consent activities will be conducted onsite at TMC. We are seeking to enroll 45 subjects for both the control and intervention group (n = 90 total). Using the National Cancer Institute screening log, the research coordinator will systematically screen all TMC patients who require palliative care for study eligibility. For patients who meet eligibility criteria, the research coordinator will introduce the study and assess interest in participation. Interested patients will be referred to the site principal investigator who is a member of the palliative care team, who will complete the informed consent process and randomize patients to the intervention or control group via computer-generated assignment. For each patient screened, the research coordinator will document study enrollment and completion outcomes (e.g. consent, enrollment, drop out, adverse events, completion).

### Randomization and blinding

Participants will be randomized to intervention or control groups via simple randomization. Allocation concealment will be used so that patient allocation assignment remains unknown until after consent. Block size will be varied to minimize the likelihood that the next treatment assignment can be guessed. Investigators and statisticians in the US will be blinded to which study arm the participants belong in to avoid bias during analysis.

### Data collection and Outcomes

To inform selection of study instruments, our team conducted a systematic review of outcome measures used in palliative care in limited resource settings [16]. Table 2 displays our study outcomes, strategies and time points for data collection, with mapping to RE-AIM Framework domains.

**Table 2:** Evaluation Measures Organized within the REAIM Framework

Domain	Outcome/Instrument	Collection, Source and Timepoints	
Outcome	Measures	Collection/Time Points	RE-AIM Domain
**Aim 1: Evaluation of Intervention Implementation**			
CHW Training/ Orientation	Attendance rates for program orientation, trainings and meetings among CHWs and clinical team; Pre/post-test change in CHW’s palliative care knowledge and perceptions; CHW’s skills performance and perceptions of training content and format post-training	Minutes from home-based palliative program meetings TMC Co-Is will administer survey pre-post training & skills checklist post-training	Adoption Implementation
CHW Role	Frequency of patient visits; assessment of patient problems (e.g. pain, transportation, depression); CHW actions (education, scheduling appt., obtaining morphine); problem resolution (pain control); frequency of debriefings w/ clinicians	Review of toolkit log data	Implementation, Adoption
Study Recruitment	Number, % and characteristics of eligible patients who are offered study participation, consent and complete/drop-out; reasons for screen failures and dropout	TMC research coordinator will complete recruitment and enrollment logs	Reach; Implementation
Stakeholder Perceptions	Perceptions of Pal-Care intervention implementation (e.g. CHW role and responsibilities, teamwork, communication, workflow; barriers, facilitators and optimal practices); intervention feasibility, acceptability and usefulness; potential for sustainability and scale up (with comparison by stakeholder type, as relevant)	Minutes from home-based palliative program meetings; Post-intervention interviews with CHWs, clinical team, patients/caregivers (§ Appendices)	Implementation, Adoption, Maintenance
Fidelity to Study Protocol	Participant recruitment per inclusion criteria; data collection per protocol (% completion of patient surveys and each type of toolkit form), regular meetings with research team/sites with high attendance; timely reporting of adverse events	Patient record reviews, review of completed surveys/toolkit forms, meeting minutes	Implementation
**Aim 2: Evaluation of Intervention Effectiveness**			
Patient Outcomes	Patient surveys (§ Appendices) will be conducted with intervention/control groups to assess palliative needs via African Palliative Outcomes Scale; [17] QOL via WHO QOL Scale; [18–20] cancer symptoms via Edmondton Symptom Scale [21, 22] and patient care experience via FAM-Care Scale [23, 24]. Palliative care toolkit forms [12] filled out by CHWs measure outcomes for intervention group: use of pain, anti-emetics and laxative medications; performance status; survival days in palliative service; location of death.	TMC research coordinator will administer surveys by phone after baseline study visit and at 1 and 3 months Review of toolkit form data	Effectiveness Effectiveness
Health System Outcomes	-Number of medical visits per patient and distribution of these visits by type (cancer center, caregiver proxy and home-based services); healthcare service costs -Navigation program continuation status 6-months post grant; Inquiries by potential navigators/organizations re: participation	Chart review of cancer center records (plus navigation logs for navigated patients) Post project qualitative interviews	Reach; Effectiveness Maintenance

The 3 primary data sources for this study include WHO Toolkit logs completed by CHWs, longitudinal surveys with intervention and control group patients/caregivers, and post-intervention stakeholder interviews. Supplemental data sources include meeting minutes, training evaluations, study recruitment logs and chart reviews of cancer center medical record/billing data. Study evaluation measures will be examined overall and by patient sex, age and cancer type and by CHW to explore potential differential intervention sub-group effects.

Surveys will be conducted with study participants at study entry (baseline) and at 1, 3, and 6 months to assess patient outcomes. The TMC research coordinator, a native Bengali speaker, will administer the baseline survey after the informed consent is completed, and will administer the follow-up surveys during patient’s routine monthly visit to TMC. As many patients have low literacy and are very sick, we selected brief instruments that use simple language and response options. Multi-dimensional palliative care outcomes will be assessed with the 10-item African Palliative Outcomes Scale that measures physical and psychological symptoms; spiritual, practical, and emotional concerns; and psychosocial needs of patient/family on a 5-point Likert scale, with higher score indicating higher symptom burden or concern [17]. Quality of life (QOL) will be assessed with the 26-item WHO QOL Scale, [18–20] that assesses physical health, psychological health, social relationships and environment on a 5-point likert scale, with higher score indicating higher QOL. Cancer symptoms will be assessed with the 9-item Edmonton Symptom Scale [21, 22]. Experience with care will be assessed with the 16-item FamCare Patient Scale measured on a 5-point likert scale [23, 24]. Table 2 below further elaborates on each of the specific evaluation of each measure. These instruments have established validity and reliability and have been used in palliative populations and limited resource settings including India, but have not been translated into Bengali. Surveys will be translated by certified translators for Hindi, Bengali, English and other Indian languages, as needed. Standard translation/back translation will be used to ensure accurate translation.

Toolkit forms: [12, 25] for data collection include: (1) a record of patients in CHW caseload; (2) a home visit record to track services, including main problems, care provided and visit notes; (3) a patient-held home care record to longitudinally document visits to patient, their condition and main problems, care provided and notes; (4) a patient-held drug chart to record name and purpose of each drug, dose and form of medication, and when each dose should be taken; (5) a patient held morphine record to document form, strength, and administration dates/times; (6) a referral form for services and resources; and (7) a monthly report to document number of patients under care, their diagnoses and contacts, new referrals, and end of care outcomes.

Post-Intervention Interviews: The Primary investigator (PI), who is a well-trained interviewer and speaks the local language Bengali and Hindi will interview stakeholders to evaluate the intervention. 20 in-person interviews will be conducted (or until saturation is reached), representing Pal-Care clinical team members (n = 6) and CHWs (n = 6) and 8 patients/caregivers who participated in the intervention. Patients/caregivers will be selected to represent experiences with different cancers, clinical problems and assigned CHWs. To fill in information gaps, additional stakeholders may be interviewed. Qualitative recommendations suggest thematic saturation is usually achieved in individual interviews with 20–30 participants [26]. We will obtain written consent and provide $10 compensation. Clinical team and CHW interviews will query how CHW’s conducted their work and engaged with patients and clinical team; training and support needed; and barriers, facilitators and optimal strategies for care delivery. Patient and caregiver interviews to be conducted in patient homes will query experiences with the CHW, education and support needed, if these needs were met, and service quality and efficiency.

A semi-structured interview guide will be designed to (1) evaluate perceptions of the Pal-Care intervention implementation/feasibility among clinical team members, CHWs and patients/caregivers, with comparisons by stakeholder type and (2) document the prominent barriers and facilitators in the delivery of the Pal-Care intervention and (3) understand the adoption of the intervention as a model for palliative care delivery in the rural communities. Probes will be used elicit clarifications and explanations to further understanding. Participants will also complete a questionnaire capturing participant demographics and attitudes related to cancer palliative care. Research questions will address the following themes within the evaluation of the Pal-Care program:

1. What are the perceptions about experiences regarding the home-based Pal-Care program?
2. What are the barriers, facilitators and resources needed to implement home-based palliative care services in the rural Indian community, including training, support? What was the impact of the COVID pandemic on service delivery?
3. What were the expectations regarding providing (for the clinical group) and receiving (for the patient/caregiver group) palliative care services and to what extent did the home-based Pal-Care program meet these expectations (e.g. satisfaction and adoption)?
4. What are the recommended strategies for improving delivery of home-based Pal-Care services within local communities?

The interview guide has been developed, tested, and refined through multiple iterations to ensure that questions are culturally appropriate and open-ended enough for participants to freely discuss what is meaningful and important to them while simultaneously eliciting information that will help evaluate the palliative care intervention.

### Data management

Based on our formative work, cancer center stakeholders prefer using hard copy patient care logs and assessment tools. The TMC research coordinator will collect the data via pen and paper and enter them (de-identified) into a password protected web-based system, REDCap. Only IRB-approved study personnel with the appropriate designations will have access to the study database. All data obtained in the study will be coded and maintained on a computer that requires an access code. All study materials will have research numbers and do not have any other identifying information. Further details on study confidentiality is provided below.

To ensure accuracy and completeness of data records, the Medical University of South Carolina (MUSC) Program Coordinator will perform bi-weekly database reviews. Considering the short-expected lifespan for participants, the bi-weekly database reviews will also increase the likelihood that any missing data can be revisited with and captured from the participant before expiration. In the event a missing or incomplete data is found during the database review, the MUSC Program Coordinator will notify the PIs and the TATA Research Coordinator. The discrepancies will be acknowledged and addressed by the TATA Research Coordinator. An Excel spreadsheet will be kept on all discrepancies found in the data to ensure acknowledgement and completeness of each discrepancy. The spreadsheet will also keep all team members abreast on database status. The PIs at the partner sites and the MUSC Program Coordinator will also ensure intervention fidelity during team de-briefing at bi-weekly meetings through videoconferencing and will monitor study data in RedCap.

### Statistical methods

Table 2 describes measures to be collected to evaluate study aims. Data will be analyzed in SAS [27]. All measures will be evaluated overall and by patient age, gender and cancer type and by CHW caseload. For aim 1, implementation measures will be reported as means, standard deviations, medians, range, frequencies and proportions as appropriate. When appropriate, outcomes will be compared between intervention and control groups using t-tests and chi-square tests (or equivalent nonparametric tests) as appropriate. For primary analyses for aim 2 the intent-to-treat (ITT) sample will be used comprising all randomized patients. Descriptive statistics will be calculated for all variables. For continuous variables we will report means, standard deviation, medians and ranges. We will compare between group differences for continuous variables using either t-test for variables that are normally distributed or can be log-transformed or a Wilcoxon rank sum test for variables if normality cannot be approximated. For categorical variables, we will report frequencies, percentages and compare between group values with Chi Square or Fisher exact test. 95% CIs will be reported. In exploratory analysis to obtain variance estimates of effectiveness outcomes and the covariance structure of the longitudinal scores, generalized linear mixed models (GLMM) will be used to compare the two groups (intervention vs. control group) with intervention group as the primary independent variable and pain as the dependent variable. GLMM can account for clustering of measurements within CHW and within patients as well as accommodate missing data. Group (intervention vs. control group) will be a fixed effect; demographics (age, sex, distance from TMC cancer center); and clinical (time since diagnosis, cancer type, baseline pain) variables will be adjusted for, along with a CHW variable accounting for cluster effects among patients by CHW. We will estimate the difference (via 95% CI) in average slopes between intervention and control groups and evaluate linearity of trajectories as input to inform a future trial. Further, dropout rate will be examined. If over 10% of data are missing, we will adjust data collection intervals in a future trial.

Partnering PIs at the Medical University of South Carolina and Clemson University will independently analyze stakeholder interviews and iteratively work together using grounded theory to develop themes using a deductive/inductive approach. Transcripts will first be reviewed to develop an initial codebook. Transcripts will be coded via constant comparison, comparing existing data with new data to refine codes. Open coding will be used to classify similar themes into categories and subcategories, which will become the basis of theoretical sampling to identify additional stakeholders for interview and modify the interview guide to fill in gaps. Stakeholder perceptions about the CHW role, responsibilities and interaction with patients and clinical team; usefulness, feasibility, acceptability and sustainability of the intervention and materials; and barriers, facilitators and strategies for optimizing the intervention, study measures and data forms will be summarized.

### Data monitoring

Per the Data Safety and Monitoring Plan for the Pal-Care intervention, the PIs at MUSC and Clemson will monitor data safety and quality. They will examine planned data records prior to beginning study and also quarterly review data records. MUSC/Clemson PIs will ensure intervention fidelity during team debriefing at bi-weekly team meetings via videoconferencing and will monitor study data in the RedCap system. If issues are identified, prompt meetings will be scheduled with the PI at TMC to develop and carry out a remediation plan. Ongoing quality control by the site PI will include regular data verification and protocol compliance checks. The research coordinator will produce monthly administrative reports that describe study progress to include accrual, demographics, and subjects’ status. These monthly reports will also identify adherence to inclusion/exclusion criteria, study protocol, adverse events. Adverse events and Protocol deviations will be monitored continuously by the PIs. All reportable Adverse Events, Serious Adverse Events and unanticipated problems experienced by participants will be reported to the NIH, respective Human Assurance Committees (MUSC IRB and TMC Ethics Review Board) in compliance with their Adverse Event Reporting Policy requirements. All protocol deviations will be reported to the respective IRBs by the Research coordinator. Data safety procedures will be part of the annual review by the appropriate IRBs and any changes suggested by these groups will be incorporated into the delineated data safety plan. A Data Safety Monitoring Board will receive reports twice a year on project progress including information on enrollment, retention, demographics, adverse events, and reportable IRB event, amendments and approvals. For the qualitative post-intervention interviews, data safety monitoring is not applicable.

### Adverse events

All adverse events will be immediately reported within 24 h to the institutional regulatory board at TATA and the team at MUSC is promptly notified thereinafter. All reportable Adverse Events, Serious Adverse Events and unanticipated problems experienced by participants will be reported to NIH, respective Human Assurance committee (MUSC IRB and TATA Ethics Review Board) in compliance with their adverse reporting policy requirements. All Adverse Events and accompanying details will be recorded in RedCap.

### Funding and participant compensation

Participants will receive $15 (approx INR 1000) incentive for participation at the time of study enrollment and again at survey data collection points (1, 3, and 6 months). The CHWs will receive a $125 monthly stipend and reimbursement for study-related costs. This study was funded by NCI/NIH R21 CA252850.

## Discussion

Dissemination and implementation of palliative care to limited resource settings has been extremely limited [2]. 80% of patients who need palliative care live in low to middle countries, but only 10% of these patients receive these services [2]. This pragmatic clinical trial will evaluate the utilization of CHWs to deliver palliative care to increase the reach of palliative care services to rural, underserved areas in India, helping to improve end of the life care for this population. Two primary study challenges are that: (1) it will be conducted in a low-resource country with primary oversight at MUSC and Clemson, and (2) CHWs without prior palliative experience may find it challenging to deliver palliative care. To overcome these challenges, several plans are in place. Proactively, we partnered with a modern cancer center with an established palliative care program, experience conducting research and modern teleconferencing capabilities. Strategies to ensure fidelity will include calls every two weeks with the TMC team, weekly meetings for CHWs to debrief with TMC team, and monitoring study data quality in RedCap. If issues are identified, we will schedule meetings with the site PI to develop a remediation plan, which may include strategies such as extra training for the TMC research team on protocols, more palliative training for CHWs or modification to the CHW role. The long-term goal of this research is to develop a feasible, effective and sustainable model for delivering home-based palliative care for cancer patients in underserved areas in rural India. If this intervention proves to be an effective strategy for improving the delivery of palliative care, it has great potential for dissemination among low to middle resource countries, as well as can likely be adaptable for use with other life-threatening illnesses beyond cancer.

### Dissemination policy

Affordable, contextually appropriate interventions are needed to bring palliative care to patients living in limited resource settings. This study leverages an existing infrastructure of CHWs who are for patients in communities across India, often representing the only rural providers. Study findings will inform future scale-up, implementation and home-based palliative care for cancer patients. Results will be disseminated through national conferences and publications and plans are in place to share the research experience adapting the Palliative Care Toolkits with the WHO, who has called for wide-scale utilization and evaluation of the toolkit in diverse global settings.

## Data Availability

The data obtained in the current study will be available from the corresponding author upon reasonable request after publication of the results on the main research questions.

## Abbreviations

AE: Adverse event
CHW: Community Health Worker
REDCap: Research electronic data capture
RCT: Randomized controlled trial
SAE: Serious adverse event
SMC: Data and Safety Monitoring Committee
SPIRIT: Standard Protocol Items: Recommendations for Interventional Trials
WHO: World Health Organization
